# Initial Experience of Articulating Laparoscopic Instruments for Benign Gynecologic Disease: A Single-Arm Multicenter Prospective Study (Korean Gynecologic Oncology Group 4002)

**DOI:** 10.3390/jpm13101433

**Published:** 2023-09-25

**Authors:** Joseph J. Noh, Kidong Kim, Tae-Joong Kim, Taehun Kim, Keun Ho Lee, Jong Ha Hwang, Dae Gy Hong, Min Kyu Kim, Banghyun Lee, Ha Kyun Chang, Kyoung Chul Chun

**Affiliations:** 1 Gynecologic Cancer Center, Department of Obstetrics and Gynecology, Samsung Medical Center, Sungkyunkwan University School of Medicine, Seoul 06351, Republic of Korea; joseph.noh@samsung.com; 2 Department of Obstetrics and Gynecology, Seoul National University Bundang Hospital, Seoul National University College of Medicine, Seongnam 13620, Republic of Korea; kidong.kim.md@gmail.com; 3 Department of Obstetrics and Gynecology, Seoul Metropolitan Government Seoul National University Boramae Medical Center, Seoul 07061, Republic of Korea; coolluck1979@gmail.com; 4 Department of Obstetrics and Gynecology, Seoul St. Mary’s Hospital, The Catholic University of Korea, Seoul 14662, Republic of Korea; hohoho@catholic.ac.kr; 5 Department of Obstetrics and Gynecology, International St. Mary’s Hospital, Catholic Kwandong University College of Medicine, Incheon 22711, Republic of Korea; jh36640@hanmail.net; 6 Department of Obstetrics and Gynecology, School of Medicine, Kyungpook National University, Daegu 41566, Republic of Korea; chssa0220@hanmail.net; 7 Division of Gynecologic Oncology, Department of Obstetrics and Gynecology, Samsung Changwon Hospital, Sungkyunkwan University School of Medicine, Changwon 51353, Republic of Korea; seoulminkyukim@gmail.com; 8 Department of Obstetrics and Gynecology, Inha University Hospital, Inha University College of Medicine, Incheon 22332, Republic of Korea; banghyun.lee@gmail.com; 9 Department of Obstetrics and Gynecology, Korea University Ansan Hospital, Korea University School of Medicine, Ansan 15355, Republic of Korea; coolblue23@naver.com; 10 Department of Obstetrics and Gynecology, Ilsan Paik Hospital, Inje University College of Medicine, Goyang 10380, Republic of Korea; kcchun@paik.ac.kr

**Keywords:** surgery, articulating laparoscopy, surgical instruments, minimally invasive surgical procedures, gynecology

## Abstract

Laparoscopic surgery has evolved with technological advances in many aspects and increasing demand for its benefits in cosmetics, fast recovery, reduced complication rates and pain. However, it still possesses drawbacks such as limited surgical movement due to the nature of rigid laparoscopic instruments. In order to overcome such limitations, several laparoscopic jointed instruments have been developed. In this prospective multicenter, single-arm cohort study, we investigated the short-term safety and feasibility of the new articulating laparoscopic instruments in benign gynecologic surgery. A total of 113 patients who were diagnosed with benign gynecologic adnexal diseases underwent laparoscopic surgery with articulating laparoscopic instruments. Surgical outcomes, including intra/postoperative complication rates, operation time and estimated blood loss, as well as surgeon’s subjective evaluation of the usage of the instruments, were evaluated. The results demonstrated that the articulating laparoscopic instruments had comparable usability and produced similar surgical outcomes to conventional laparoscopic surgery. The objective parameters, such as the operative time and complication rates, as well as the subjective parameters, such as the surgeon’s own evaluation of the surgical instruments’ usability, demonstrated potential benefits of the instruments in benign gynecological diseases. Overall, the study demonstrated that the use of this novel articulating device is feasible in gynecologic laparoscopic surgery.

## 1. Introduction

With increasing demand for minimally-invasive surgery (MIS) in the gynecologic field, laparoscopic surgery has been widely implemented for its benefits in the treatment of benign and malignant disease [1,2,3]. MIS causes less trauma and has been shown to provide a faster postoperative recovery. However, the limited range of motion of the laparoscopic instruments within the abdominal cavity remains a challenge. Recently, robotic surgery has gained increasing acceptance and popularity in this field due to its considerably improved vision with a 3D view, enhanced dexterity, greater precision and easier performance of procedures [4,5]. However, the high cost of the robotic platform and its limited availability have impeded its implementation in many regions, and the conventional laparoscopic technique remains a frequently performed surgical method that accounts for a significant portion of patient care [6,7,8].

Laparoscopic surgery has evolved with technological advances in many aspects. However, these surgical methods still have several drawbacks. Among them are the lack of tactile sensation and limitations in movement due to the nature of rigid laparoscopic instruments [9,10,11,12,13]. In this study, we introduce new laparoscopic instruments, called ArtiSential^TM^, with articulating arms that may enable surgeons to overcome some of those limitations. These instruments comprise a complete line up of mechanical, hand-held and single-use laparoscopic instruments. They transfer the motion of the surgeon’s finger movements to the end effector directly. The device has been used in the clinical setting since 2019 after approval by the Ministry of Food and Drug Safety of Korea and the Health Insurance Review and Assessment Service of Korea. Because its usage in the clinical field is relatively new in gynecologic surgery, there have been only a few reports on its clinical usefulness [14,15,16,17,18,19,20,21,22,23].

In this prospective multicenter single-arm cohort study, we investigated the short-term safety and feasibility of ArtiSential^TM^ in adnexal surgery. This study was conducted at 10 expert centers that perform at least 300 cases of minimally invasive surgery per year to treat benign and malignant gynecologic disease.

## 2. Materials and Methods

The present study was conducted as a prospective multicenter cohort study by the Korean Gynecologic Oncology Group (KGOG). The KGOG has conducted several studies evaluating the feasibility and safety of the articulating laparoscopic instruments in various surgical settings: adnexal surgery, hysterectomy, myomectomy and cancer surgery. The present study was one of the above several investigations, and the surgical outcomes from adnexal surgery are reported here. Results from hysterectomy, myomectomy and cancer surgery will be reported separately in other studies. The present study included patients who were diagnosed with benign gynecological adnexal disease and scheduled for surgical treatment at 10 academic tertiary medical centers in South Korea between November 2021 and March 2022. The surgeons included in the present study were all experienced in minimally invasive surgery in gynecology. However, they had not used any articulating laparoscopic instruments prior to their participation in the study. At the beginning of the study, therefore, all the surgeons were provided with training boxes so that they could practice how to manipulate and handle the instruments. Surgeon competency with the instruments was not assessed in the study. They were allowed to start using the instruments whenever they felt comfortable using them. Adnexal disease was defined as one or more of the following: ovarian cyst which required cystectomy or oophorectomy, salpingeal disease such as salpingitis, hydrosalpinx, tubal abscess or tubo-ovarian endometriosis that required salpingectomy and/or any other procedures that involved salpingo-oophorectomy. Patients who required uterine surgery, such as myomectomy, adenomyomectomy or hysterectomy, were not included.

**Figure 1:**
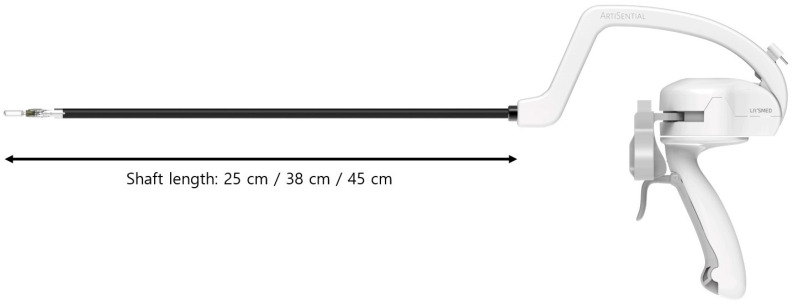
Artisential^TM^ articulating laparoscopic instrument.

Before surgery, a detailed collection of previous medical/operative history, related symptoms and imaging results, including ultrasonography, computed tomography (CT), or magnetic resonance imaging (MRI), were first reviewed. For those patients who decided to undergo surgical procedures, attending surgeons explained about the articulating laparoscopic instruments and obtained patients’ informed consent to proceed with the study. The new laparoscopic articulating instruments (ArtiSential^TM^, LIVSMED Inc., Seongnam, Republic of Korea) have an 8 mm and 5 mm shaft and provide 360° of free motion of the end-effector in robotic surgery by controlling two pinching triggers on the handle (Figure 1). Video clips of simple manipulation of the articulating laparoscopic instrument are provided in the Supplementary Video S1. The instruments come in three different shaft lengths: 25 cm, 38 cm and 45 cm. Depending on the type of surgery and entry site location, a surgeon can choose his or her instrument that provides the best ergonomic positions. These instruments were registered as Class I medical devices with the Food and Drug Administration in 2019 and became available in clinical settings from 2019. The ArtiSential articulating laparoscopic instruments are provided in various types including bipolar fenestrated forceps, bipolar Maryland dissector, needle holder, clip applier, monopolar spatula and monopolar hook.

All the patients underwent the same standard preparation before surgery. Prophylactic antibiotics were administered 30 min before incision. After general anesthesia and endotracheal intubation, the patients were placed in the Trendelenburg position with lithotomy. The exact number and location of ports and/or trocars were determined by the attending surgeons. CO_2_ gas pressure, length and type of endoscopy camera, laparoscopic surgical instruments and advanced energy devices were all determined by the attending surgeons as well.

The baseline characteristics of the patients, including the results of the preoperative imaging studies, were collected. Outcome measurements included patient age, body mass index (BMI), previous history of abdominal surgery, adnexal tumor size, surgical indications, final pathology, total operative time, estimated blood loss (EBL), hemoglobin changes between preoperative and postoperative status, number, type and location of laparoscopic ports/trocars, intraoperative complications such as bowel, ureter and bladder injury, and immediate postoperative complications (occurring within 30 days of surgery) including ileus, wound dehiscence, surgical site infection, and transfusion. The total operative time was defined as the time from the beginning of the skin incision until the completion of skin closure. EBL was calculated by subtracting the volume of irrigating fluid from the volume of total fluid collected in the suction apparatus after surgery. Hemoglobin level change was the difference between preoperative and postoperative hemoglobin levels. Postoperative hemoglobin was measured within 24 h after surgery. The tumor size was measured before surgery by ultrasonography, CT, or MRI.

The data are expressed as mean ± standard deviation for continuous variables. Statistical significance was determined using Fisher’s exact test for dichotomous variables and the independent Student’s *t*-test for continuous variables. Statistical significance was set at *p* < 0.05. Statistical calculations were performed using R version 3.6.2. (Vienna, Austria; http://www.R-project.org/, accessed on 30 June 2023.). All procedures were performed in accordance with the ethical standards of the institution and with the 1964 Helsinki Declaration (and its later amendments). Approvals were obtained from each institution’s own Institutional Review Board.

## 3. Results

A total of 128 patients from 10 institutions were initially enrolled in the study. Out of those, 14 patients were excluded from the final analysis because the surgeons did not use any articulating laparoscopic instruments at all during surgical procedures. One patient was excluded because she withdrew her consent just prior to her surgery. Therefore, a total of 113 patients were included in the final analysis.

**Figure 2:**
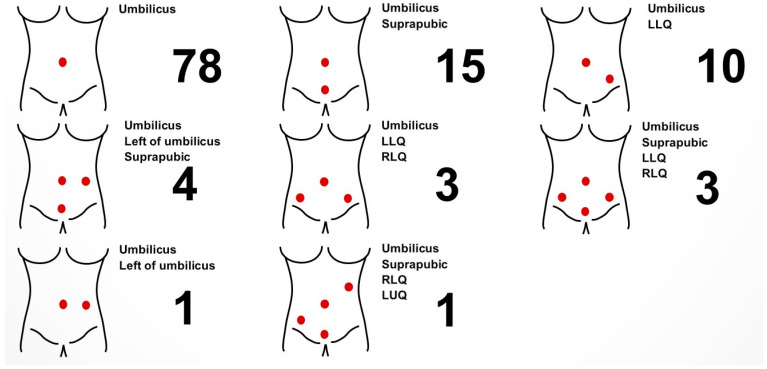
Number of patients according to different location of ports/trocars used. LLQ: left lower quadrant, RLQ: right lower quadrant.

**Figure 3:**
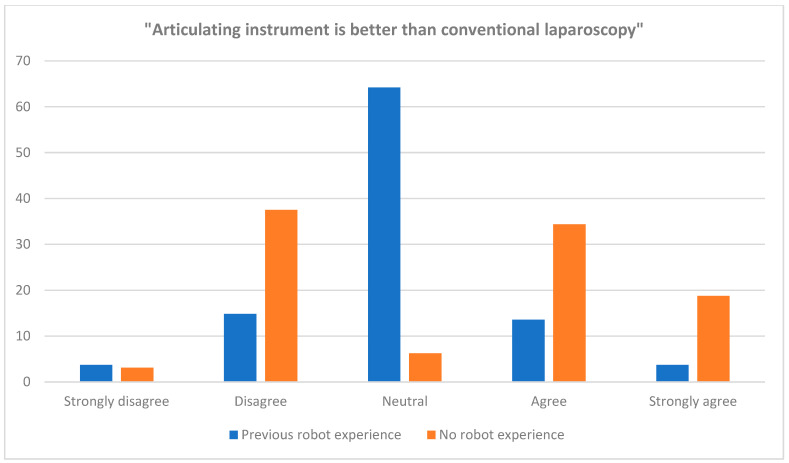
Surgeons’ answers to “Do you think the articulating laparoscopy is better than conventional laparoscopy?”.

A summary of demographics and clinical characteristics is described in Table 1. The mean age of the patients was 45 years old. About 61% of the patients had no history of previous abdominal surgery while the rest had received at least one abdominal surgical procedure prior to enrollment in the present study. Surgical information is shown in Table 2. While most surgeons used articulating laparoscopic instruments in conventional laparoscopic surgeries (96.5%), four surgeons used articulating laparoscopic instruments when performing robot-assisted laparoscopic surgeries as an assist arm in addition to robotic arms (3.5%). The exact number and location of ports and/or trocars, as well as the type of instrument used, were determined by the attending surgeons (Figure 2 and Supplementary Table S1). In most cases, operators were standing on the left side of their patients, while in some procedures, such as sacrocolpopexy (performed along with adnexal surgery), omentectomy and left adnexal surgery, the main surgeons stood on the right side of their patients. About two-thirds of the surgeons reported that they used articulating laparoscopic instruments only partially during the entire procedure while about one-thirds reported that they used them throughout the entire surgical procedure. A total of 70 cases were categorized as salpingo-oophorectomy while 36 cases were reported to be ovarian cystectomy (Table 3). Surgical outcomes are shown in Table 4. The mean total operation time was 66 min. No case was converted to laparotomy. The mean hemoglobin change between before and after surgery was −1.40 g/dL. Two patients received two packs of red blood cell transfusions postoperatively due to blood loss and none received platelet or plasma transfusion. The two patients who received transfusions were identified as those who had low hemoglobin levels prior to surgery. One patient had a hemoglobin level of 7.7 g/dL and another patient had a hemoglobin level of 8.2 g/dL. Those two patients underwent ovarian cystectomy for endometriosis. As is often the case for endometriosis, those two patients had severe adhesion around the uterus and had to undergo an extensive adhesiolysis along with their cystectomy. During the adhesiolysis, blood oozing from the uterus, pelvic peritoneum and adjacent structures caused a moderate amount of blood loss, which eventually required them to receive blood transfusions. The median diameter of excised mass from surgery was about 6 cm, with the largest being 19 cm. One patient had small bowel serosa injury during her surgical procedure, which was immediately repaired intraoperatively and had no subsequent complication. The injury was not caused by the articulating laparoscopic instrument, however, but was caused by another conventional laparoscopic instrument that was used concurrently. No conversions to laparotomy were recorded. At the end of each surgery, participating surgeons evaluated each surgical case and answered a pre-distributed questionnaire. The proportion of the surgeons who had experience of robot-assisted laparoscopic surgery before the present study was about 72%, while the rest had not had any opportunity to perform prior robotic surgery. When asked whether the articulating laparoscopic instruments were better than conventional laparoscopic instruments (rigid instruments), the surgeons who did not have any experience in robotic surgery tended to answer more favorably compared to those who had already experienced the robotic surgical platform (Table 5 and Figure 3). However, this difference was not statistically significant between the groups.

## 4. Discussion

The present study demonstrated that the new articulating laparoscopic instruments may provide solutions to overcome the disadvantages of using conventional laparoscopic instruments with fixed joints, such as reduced dexterity, limited range of movement and uncomfortable ergonomics. It demonstrated that the articulating laparoscopic instruments had comparable usability and produced similar surgical outcomes to conventional laparoscopic surgery. The objective parameters, such as the operative time and complication rates, as well as the subjective parameters, such as the surgeon’s own evaluation of the surgical instruments’ usability, demonstrated potential benefits of the instruments in benign gynecological diseases.

In gynecologic surgery, laparoscopic surgery is rapidly replacing open surgery due to its positive perioperative outcomes, and surgical instruments are being developed accordingly. However, despite the fact that laparoscopic surgery is currently popular in the field of gynecologic surgery, this procedure is still challenging. The robotic surgical platform may address some of the limitations of conventional laparoscopic instruments. However, the high cost still impedes its wide acceptance and implementation in clinical settings [24,25,26,27]. A robotic surgical system offers multi-jointed instruments, enhanced ergonomics and 3D vision. However, robot-assisted surgery is considerably more expensive than laparoscopic surgery. Therefore, in many regions of the world, robotic surgery is not available or is only available for those who can afford it. The exact costs for different surgical platforms—conventional laparoscopic surgery, robotic surgery and open surgery—all differ by region. Therefore, it is difficult to directly compare the economic benefits that the articulating laparoscopic instruments may provide. However, it is generally accepted by all healthcare professionals that the robotic surgical platform costs significantly more than the conventional laparoscopic surgical platform, and therefore it is hard to deny the financial benefits the articulating laparoscopic instruments provide in comparison to the conventional laparoscopic settings. Several laparoscopic jointed instruments have been developed as alternatives to the costly robotic systems. The articulating joints of the instruments are synchronized with the surgeon’s wrist motion. Compared to conventional laparoscopic instruments, these new instruments provide easier movement for surgical procedures, especially in narrow anatomic spaces. Considering the fact that these single-use articulating laparoscopic instruments are readily available with diverse line ups, advantages in everyday surgical practice, especially where robotic surgery is not available, can be anticipated. Conventional straight laparoscopic instruments have the disadvantages of reduced dexterity and limited movement. Such limitations become more prominent in pelvic surgery with large tumors. It becomes challenging for surgeons to obtain an effective traction or angle approach during conventional laparoscopic surgery. The articulating laparoscopic instruments used in the present study help surgeons easily obtain effective traction through intuitive movements. The instruments are fully synchronized with the surgeon’s hand and can be moved in a 360° arc, allowing for more versatile surgical procedures. The handle grip itself is constructed in such a way as to allow for movement of the hand in the horizontal and in the vertical plane [22]. Adding bidirectional rotation of the hand along the axis of the forearm, a 360° hemispherical space of motion becomes available for the end effector [22]. This basically eliminates the fulcrum effect principle, known from standard laparoscopy. It also allows surgeons to have good surgical views during surgery. In single-port access laparoscopic surgery, the camera and laparoscopic instruments are usually placed parallel to each other, forming a straight line. This formation sometimes blocks the laparoscopic camera’s view. By using articulating laparoscopic instruments, surgeons can bend instrument tips and can make effective surgical maneuvers without blocking the camera view. Images and video clips of actual surgical procedures are provided in Supplementary Figure S1 and Supplementary Video S2. These single-use, low-priced articulating laparoscopic instruments may reproduce the advantages of the robotic surgery in a laparoscopic setting. This novel articulating instrument may be a good alternative to robotic surgical systems, with the same clinical benefit but reduced overall cost.

It is hard to deny that robotic surgery is more ergonomically favorable than laparoscopic surgery and this fact has promoted its further clinical use in many surgical specialties. Robotic systems have improved visualization and provide enhanced manipulation of tissue by allowing wrist-like movements of instruments. However, robotic surgery does not provide touch sensation, which may play an important role in delicate adnexal surgical procedures such as those procedures for deep infiltrating endometriosis. Conventional laparoscopic instruments, on the other hand, allow surgeons to feel the hardness of grasping tissue. It also allows the operating surgeons to feel the strength of tissue when applying force for traction or counter traction. The articulating laparoscopic instruments used in the present study also allow surgeons to feel the tissue they are manipulating, therefore enabling them to proceed with delicate and meticulous surgical procedures.

One notable finding of the present study is the fact a significant portion of the surgeons used articulating laparoscopic instruments in single-port access surgery. About 70% of the surgical cases were performed by single-port access laparoscopy (78 out of 113 cases). One of the major limitations of single-port access surgery is frequent extracorporeal instrument collision and thereby reduced maneuverability. Despite the fact that the articulating laparoscopic instruments in the present study have more bulky handles than conventional laparoscopic instruments, more than two-thirds of the cases were performed by single-port access, implying the usability of the devices in narrow spaces as well. It is also notable that, in four cases, surgeons used articulating laparoscopic instruments in their robotic surgical cases. In such cases, the instruments were used as an assistant instrument rather than the main surgical instrument. The instruments come in various types including scissors, graspers, spatula and needle driver, among many others. The availability of various types of instrument allows their usage in diverse conditions, not only as the main surgical instrument but also as a useful assistant instrument.

A significant portion of the surgeons in the present study used the articulating laparoscopic instruments in single-port access laparoscopic surgery. Single-port access laparoscopy has been widely implemented for a variety of gynecologic diseases. Despite the safety and feasibility of this surgical platform, challenges still remain. The technical and ergonomic difficulties of single-port access surgery include limited triangulation technique and poor visualization. Moreover, the steep learning curve is one of the major obstacles for surgeons to become proficient in single-port access laparoscopic surgery. Robotic single-site surgery has gained increasing acceptance and popularity in the field of gynecologic surgery as a means to overcome such limitations. It provides more comfortable ergonomics for surgeons with a wider range of motion, and improved dexterity and camera stability. It also has less instrument crowding. Despite these advantages, several drawbacks of the robotic single-site platform still exist, including semi-rigid robotic instruments that are not wristed at the tip like multiport robotic platform instruments. Semi-rigid robotic instruments are weak and make it difficult for surgeons to generate enough grasping power for traction. Articulating laparoscopic instruments may provide solutions to overcome the limitations of each surgical approach. They have rigid instrument arms that are wristed at the tip, providing adequate triangulation. They also allow surgeons to perform surgical procedures on the single-port access platform but at the same time provide intuitive instrument maneuvers like robotic instruments.

Another important issue to note is the availability of advanced surgical energy devices. In surgery with articulating laparoscopic instruments, surgeons can freely use any preferred advanced surgical energy device. However, the use of advanced surgical energy devices is relatively limited in robotic surgery. There are not as many options for energy devices in robot-assisted laparoscopic surgery as there are in conventional laparoscopic surgery. Articulating laparoscopic instruments may allow surgeons to benefit from their robot-like movement while allowing the use of any preferred advanced energy devices at the same time.

The use of articulating laparoscopic instruments carries its own limitations in the present form, however. First, they are slightly bigger and heavier than conventional straight laparoscopic instruments and therefore surgeons with small hands could have difficulties to handle them. A surgeon may experience physical stress from the heavier instrument compared to the conventional instruments; they have to bear the weight on their wrists during the entire surgical procedure. The instrument’s proximal body is also bulky because it contains rotating apparatus in it, which may increase instrument collisions if more than one instrument is inserted into the same entry site. These issues may adversely affect the introduction of the articulating laparoscopic instruments. Improved devices are currently being developed to overcome such limitations, with lighter handles and smaller body parts. Another limitation of the articulating laparoscopic instruments used in the present study is that they required a certain amount of learning on the part of practicing surgeons. Although most surgeons eventually became comfortable using the instruments in their actual surgical settings without significant difficulties, they had to learn the basic movements of the instruments by practicing in the training boxes (Supplementary Figure S2). Future studies are suggested to assess the learning curve of the articulating laparoscopic instruments, conventional laparoscopic instruments and the robotic surgical platform.

The present study has the following limitations. First, the study did not include a control arm (conventional laparoscopic surgery) to compare operative outcomes. Because the present study was conducted at 10 different academic institutions, variation in patient selection, detailed surgical techniques, and perioperative care inevitably exists. However, despite the fact that the present study was conducted as a multicenter, single-arm study, the surgical outcomes observed were similar to those seen in previous studies reported in the literature [28,29,30], indicating that the use of articulating instruments may be safe and feasible, without increasing operation time or intraoperative/postoperative complications. A Korean group previously reported their first 200 cases of single-port access surgery in gynecology in 2010 [28]. In that report, the median operation time was 60 min (range 27–245 min) for oophorectomy, 105 min (range 50–185 min) for cystectomy and 60 min (range 30–115 min) for salpingectomy. The median estimated blood loss was 30 mL (range 10–400 mL) for oophorectomy, 50 mL (range 30–500 mL) for cystectomy and 70 mL (range 10–100 mL) for salpingectomy. Although it may not be appropriate to directly compare the surgical outcomes of the present study to what were seen in the previous study because of the different patient populations, the surgical outcomes observed in the present study do not seem significantly different. However, a larger comparative study that evaluates the benefits of the articulating laparoscopic instruments compared with conventional laparoscopic instruments or robot-assisted laparoscopic surgery is suggested. Second, this study did not include any learning curve analysis. Although previous studies have reported a trend towards decreasing times to perform specific surgical procedures with articulating laparoscopic instruments, as well as decreasing total operative times, further studies are required to objectively assess the learning curve in the usage of these instruments in gynecologic surgery [20]. The surgeons in the present study were all experienced surgeons, with many years of experience in either conventional laparoscopic surgery or in both conventional laparoscopic surgery and robotic surgery. Therefore, the perioperative outcomes obtained in the present study may not be generalizable to other clinical settings with less experienced surgeons. Third, most surgeons in the present study used the articulating laparoscopic instruments in single-port access surgery. Therefore, this limits the generalizability of the surgeons’ opinions observed in the study to other surgical platforms. It should also be noted that the questionnaire used in the present study, which contained four subjective questions, was developed by the researchers of the present investigation. The articulating laparoscopic instruments are relatively new in the field of gynecologic surgery, therefore further studies are needed to assess surgeons’ subjective opinions on the usability of these instruments with a more detailed and validated questionnaire. Last, the study analysis was retrospective despite the prospective nature of patient enrollment and data collection.

In summary, this study demonstrated that the use of this novel articulating device was feasible in gynecologic laparoscopic surgery. More studies are needed to determine the usefulness of these instruments for certain indications. Improved articulating devices may overcome the limitations of the current devices for laparoscopic surgery. Comparative and randomized trials are suggested to elaborate more on the results of the present study.

## 5. Conclusions

The articulating laparoscopic instruments have joint structures that are synchronized with the movements of the surgeon’s hands. They allow a wider range of movement of the instrument tip, providing greater access to, and robot-like movement in, narrow surgical sites. These instruments have already been introduced in other surgical specialties and have received positive feedback. However, the feasibility and safety of these instruments have not been studied in the field of gynecologic surgery. The results of the present study demonstrated that the use of these instruments in adnexal surgery for benign gynecological disease is feasible, safe and produces comparable surgical outcomes without increased complications rates.

## Figures and Tables

**Table 1:** Baseline characteristics of the patients.

	Values (N = 113)
Age (years)	45 (18–85)
Height (cm)	159.98 ± 6.99
Weight (kg)	59.65 ± 10.59
BMI * (kg/m^2^)	23.31 ± 3.86
Number of previous abdominal surgeries None 1 2 3	69 (61.1%) 31 (27.4%) 11 (9.7%) 2 (1.8%)
Type of previous abdominal surgery Cesarean section Hysterectomy Other gynecologic surgeries Appendectomy Others	24 (21.2%) 8 (7.1%) 10 (8.8%) 9 (7.9%) 5 (4.4%)
Comorbidity Hypertension Diabetes mellitus Dyslipidemia Thyroid disease Others	53 (46.9%) 24 (21.2%) 38 (33.6%) 20 (17.7%) 12 (10.6%)

* BMI: body mass index.

**Table 2:** Surgical information.

	Values (N = 113)
Type of approach Conventional laparoscopy Robot-assisted	109 (96.5%) 4 (3.5%)
Laterality Unilateral Left Right Bilateral	36 (31.9%) 23 (20.4%) 50 (44.2%)
Operator position Left Right	109 (96.5%) 4 * (3.5%)
Type of camera No angle 30-degree angled 45-degree angled Flexible camera	9 (8.0%) 101 (89.4%) 0 3 (2.7%)
Proportion of instrument use Almost all Partially Almost none	34 (30.1%) 77 (68.1%) 2 (1.8%)

* One robot surgery, one sacrocolpopexy, one omentectomy and one left adnexal surgery.

**Table 3:** Types of surgery.

	Cystectomy (N = 36)	Salpingectomy (N = 3)	Salpingo-Oophorectomy (N = 70)
Unilateral Left Right	21 11	1 0	14 12
Bilateral	4	2	44

**Table 4:** Surgical outcomes.

	Values (N = 113)
Operation time (minutes)	66 (28–223)
Conversion to laparotomy No Yes	113 (100%) 0
Hemoglobin change * (g/dL)	−1.40 ± 1.04
Number of RBC * pack transfusions 0 1 2 ≥3	111 (98.2%) 0 2 (1.8%) 0
Number of platelet concentrate pack transfusions 0 1 ≥2	113 (100%) 0 0
Number of FFP * pack transfusions 0 1 ≥2	113 (100%) 0 0
Number of postoperative hospital stays (days) 0 1 2 3 4 ≥5	4 (3.5%) 57 (50.4%) 33 (29.2%) 9 (8.0%) 2 (1.8%) 8 (7.1%)
Pathologic diagnosis Benign ovarian cyst Endometriosis Mature cystic teratoma Tubo-ovarian abscess Others	79 (69.9%) 16 (14.1%) 9 (8.0%) 4 (3.5%) 5 (4.4%)
Size of the excised mass (cm)	6 (1–19)
Operative complication No Yes	112 (99.1%) 1 ^#^ (0.9%)

* Hemoglobin change: postoperative hemoglobin minus preoperative hemoglobin, RBC: red blood cell, FFP: fresh frozen plasma. ^#^ Small intestine injury.

**Table 5:** Operators’ opinion.

	Values
Have you performed the same type of surgery with conventional laparoscopy? No Yes	0 113 (100%)
Do you think the articulating laparoscopy is better than conventional laparoscopy? Strongly agree (articulating instrument is better) Agree They are similar Disagree Strongly disagree (articulating instrument is worse)	9 (8.0%) 22 (19.5%) 54 (47.8%) 24 (21.2%) 4 (3.5%)
Have you performed the same type of surgery with robot-assisted laparoscopy? No Yes	32 (28.3%) 81 (71.7%)
Do you think the articulating laparoscopy is better than robot-assisted laparoscopy? (Out of 81 surgeons who have robot surgery experience) Strongly agree (articulating instrument is better) Agree They are similar Disagree Strongly disagree (articulating instrument is worse)	0 1 (1.2%) 57 (70.4%) 16 (19.8%) 7 (8.6%)

## Data Availability

The data presented in this study are available on request from the corresponding author. The data are not publicly available due to patient privacy.

## Supplementary Materials

The following supporting information can be downloaded at: https://drive.google.com/file/d/1qTqQ65ul8T3zeCTIVAT6Fuv-V-uC8Kdj/view?usp=sharing (accessed on 8 September 2023), Table S1: Number of cases according to the types of instruments and their entry points, Figure S1: Actual images of surgical procedures by the articulating laparoscopic instruments, Figure S2: Training boxes offered to the surgeons to practice the articulating laparoscopic instruments, Video S1: Simple manipulation of the articulating laparoscopic instruments in a training box, Video S2: Video clips of surgical procedures performed by the articulating laparoscopic instruments.
