# Supplementary material for: Initial Experience of Articulating Laparoscopic Instruments for Benign Gynecologic Disease: A Single-Arm Multicenter Prospective Study (Korean Gynecologic Oncology Group 4002)
Source: J Pers Med. 2023 Sep 25;13(10):1433. doi: 10.3390/jpm13101433 (PMC10608162; doi:10.3390/jpm13101433)
Supplement: Supplementary file 1 [file jpm-13-01433-s001.zip › jpm-2598563-supplementary.pdf]

Supplementary files link:
https://drive.google.com/file/d/1qTqQ65ul8T3zeCTIVAT6Fuv-V-uC8Kdj/view?usp=sharing
